# Alumanyl Reduction, Reductive Coupling and C–H Isomerization of Organic Nitriles

**DOI:** 10.1021/acs.organomet.4c00289

**Published:** 2024-08-16

**Authors:** Henry T. W. Shere, Han-Ying Liu, Michael S. Hill, Mary F. Mahon

**Affiliations:** Department of Chemistry, University of Bath, Claverton Down, Bath BA2 7AY, U.K.

## Abstract

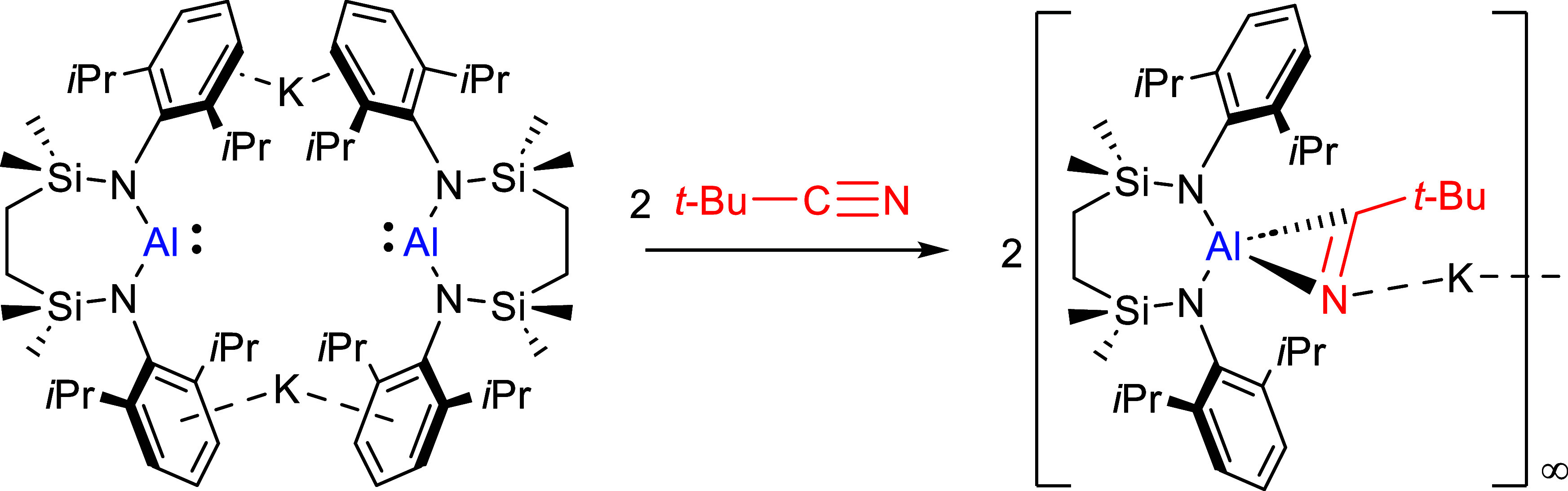

The behavior of the potassium alumanyl, [{SiN^Dipp^}AlK]_2_ ({SiN^Dipp^} = {CH_2_SiMe_2_N(Dipp)}_2_; Dipp = 2,6-*i*-Pr_2_C_6_H_3_), toward organic nitriles has been investigated. In common with earlier studies of the reactivity of charge neutral Al(I) species with multiply bonded small molecules, it is suggested that the initial step in all the reactions involves [2 + 1] cycloaddition and the generation of an [η^2^–C=N–Al] alumina azacyclopropane unit. In the cases of *o*- and *m*-tolyl-substituted aryl nitriles, this species is too kinetically labile to allow its isolation and undergoes C–C coupling via immediate Al–C/C≡N insertion to yield the alumina diazabutadiene derivatives. In contrast, the increased steric profile of alkyl nitriles imposes a marked influence on the nature of the products formed. Consistent with the proposed sequential pathway, reaction of [{SiN^Dipp^}AlK]_2_ with *t*-BuCN provides an isolable alumina cyclopropane species that is kinetically resistant to onward reaction with a further nitrile equivalent. While reduction in the alkyl nitrile steric demands by use of *i*-PrCN again facilitates C–C bond formation, the crowding of the Al center by the resultant alumina-diazabutadienediide moiety appears to be beyond the limit of kinetic viability, resulting in an unusual 2-fold C–H to N–H isomerization from one of the *C-*iso-propyl substituents and the isolation of a 1-alumina-2,5-diazabutadiene structure.

## Introduction

**Figure 1:**
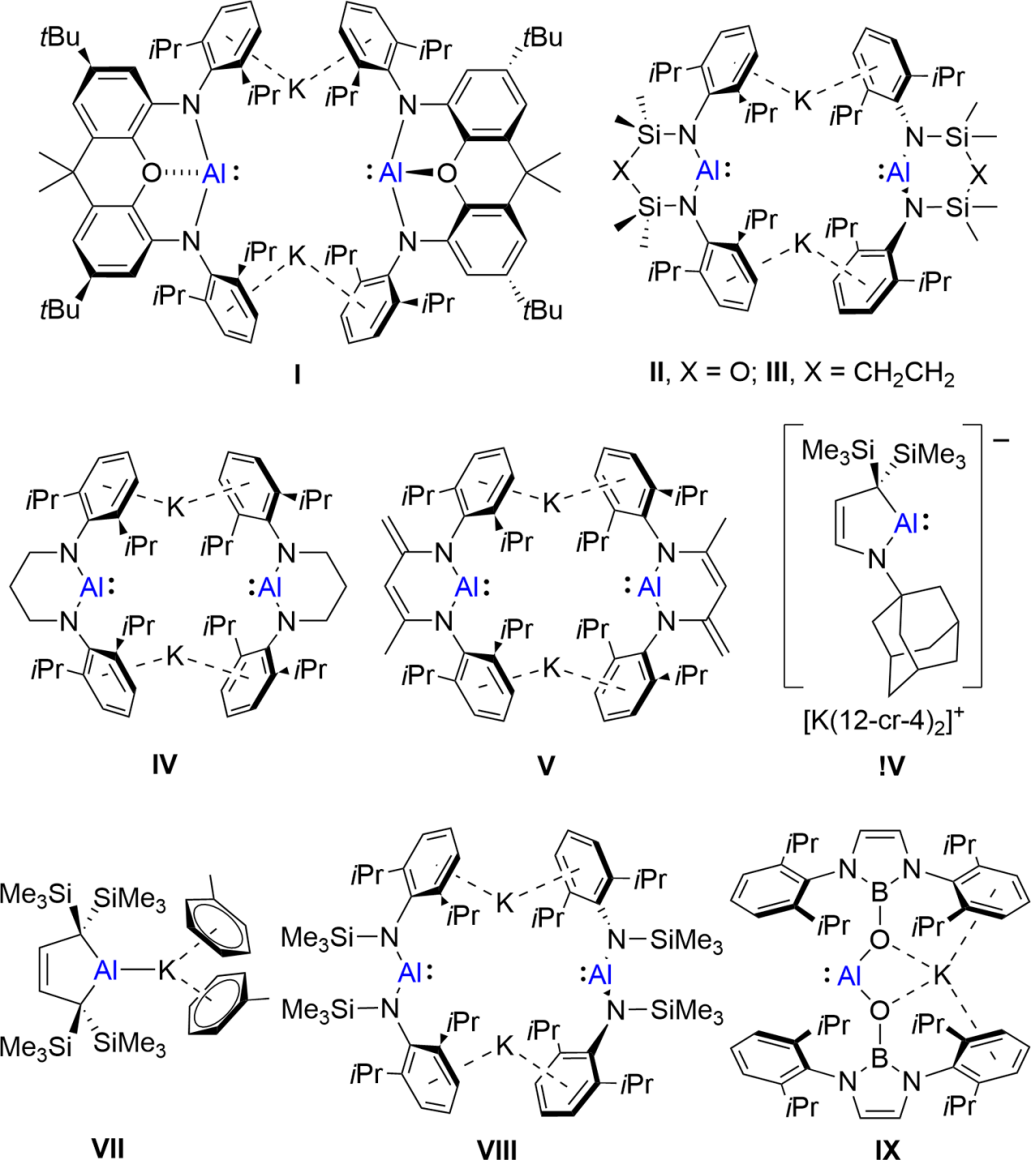
Exemplary cyclic and acyclic potassium alumanyl compounds.

Aldridge, Goicoechea and co-workers’ 2018 synthesis of the potassium alumanyl derivative, [K{Al(_xanth_NON)]_2_ (**I**, _xanth_NON = [4,5-(NDipp)_2_-2,7-*t*-Bu_2_-9,9-Me_2_-xanthene]^2–^ where Dipp = 2,6-*i*-Pr_2_C_6_H_3_; Figure 1),^1^ initiated a significant new area of main group element synthesis. The intervening 6 years have seen the development of a slew of closely related cyclic diamido-, amidoalkyl- and dialkylalumanyl derivatives (e.g., **II**–**VII**, Figure 1).^2−7^ More recent efforts have extended such potassium-containing systems to acyclic species (e.g., **VIII**, **IX**, Figure 1),^8,9^ which can exist in both contact ion pair and charge separated forms. A number of examples comprising alternative alkali metals are also known, and the structural and electronic impacts of this further variable are now beginning to emerge.^10−12^ The combination of a formally anionic Al(I) center and a Lewis acidic group 1 cation has given rise to a panoply of reactivity in which the aluminum anion acts either as a counterintuitive nucleophile or a high energy reservoir of two electrons that are available for the reduction and activation of both inorganic or organic small molecules.^13^

While this reactivity already encompasses a variety of challenging bond activation and bond-forming processes, and notwithstanding notable behavior toward arenes,^1,7,9−12,14^ alkenes,^2,15^ diazoalkanes,^6b,16^ isonitriles,^17^ ketones^18^ and carbodiimides,^3^ studies of alumanyl behavior toward many of even the most common organic functions remain unrepresented. This situation is in significant contrast to, say, the now extensive reports of the use of low oxidation state magnesium derivatives, an element that fulfils similar criteria in terms of earth abundance and supply, and which have even been designated as potential “quasi-universal” hydrocarbon-soluble reducing agents in organic synthesis.^19^ For instance, a variety of C–C and N–N bond-forming reactions are known to be induced by Mg(I) reagents in conjunction with organic isonitriles, isocyanates, alkyl azides and nitriles.^20−22^

**Scheme 1:**
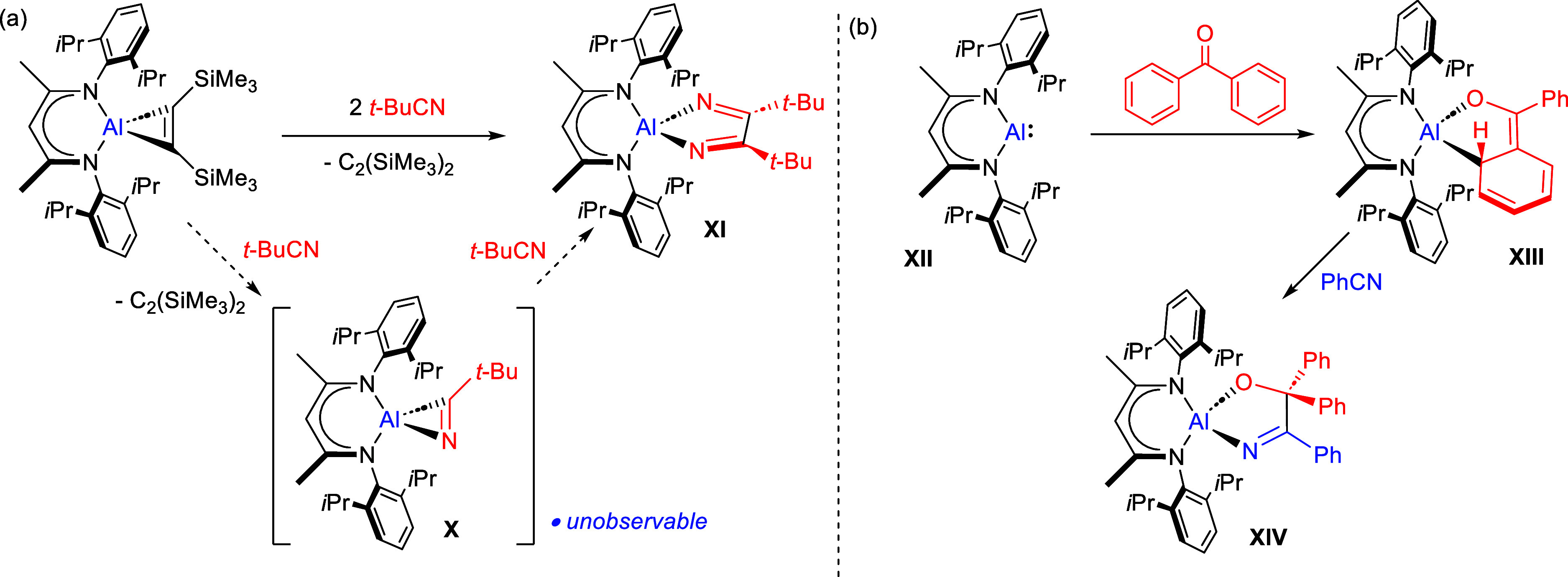
(a) Roesky and Co-workers’ Proposed Route to Compound **XI** via the Proposed Intermediate **X**;^23^ (b) Nikonov and Co-workers’ Synthesis of **XIV** via the Isolable Ketylate Derivative **XIII**(24)

In the specific case of aluminum-centered nitrile reduction, the most direct precedent is provided by Roesky and co-workers’ report of the reaction of the β-diketiminato alumina cyclopropene derivative, [{HC(C(Me)NDipp)_2_}Al{η^2^-C_2_(SiMe_3_)_2_}], with *t*-BuCN (Scheme 1a).^23^ This transformation was proposed to occur through the displacement of Me_3_SiC≡CSiMe_3_ and the formation of an unobservable aluminum η^2^-nitrile intermediate (**X**), which is immediately consumed by reaction with a second nitrile molecule to provide the aluminum diazabutadiene derivative (**XI**). Although the connectivity of **XI** was established by a poor quality X-ray diffraction analysis, no further mechanistic information has ensued. This reactivity, however, is strongly reminiscent of Nikonov and co-workers’ more recent utilization of the neutral Al(I) precursor to Roesky’s alumina cyclopropene, [{HC(C(Me)NDipp)_2_}Al] (**XII**), to effect the stepwise assembly of, among other compounds, the alkoxy-imido species (**XIV**) through the initial formation of the aluminum ketylate (**XIII**) (Scheme 1b).^24^ The C–C coupling processes exemplified by the formation of **XI** and **XIV** bear a superficial resemblance to the products resulting from the single electron intermediates generated during a classical pinacol coupling process.^25^ The formation of these compounds and the intermediate species **X** and **XIII**, however, appear better considered as a series of concerted two electron processes as has been convincingly deduced for the related [2 + 1] cycloaddition of C=C multiple bonds to the aluminum(I) centers of molecules such as **XII**.^26^

Reminiscent of Nikonov’s observation of the reactivity of **XII**, we have previously observed that [{SiN^Dipp^}AlK]_2_ (**III**, {SiN^Dipp^} = {CH_2_SiMe_2_N(Dipp)}_2_; Figure 1) displays diverse reactivity toward carbonyl reagents that, dependent on the identity of the ketone substituents, can give rise to related C–C coupled products.^18a^ Elaborating on these observations, therefore, in this contribution we extend our study of the reactivity of compound **III** toward organic nitriles.

## Experimental Section

### General Considerations

All manipulations were carried out using standard Schlenk line and glovebox techniques under an inert atmosphere of argon. NMR experiments were conducted in J Young’s tap NMR tubes prepared and sealed in a glovebox. NMR spectra were recorded on a Bruker AV-400 (400 MHz) spectrometer or an Agilent ProPulse spectrometer operating at 500 MHz and at a temperature of 298 K unless stated otherwise. The spectra were referenced relative to residual protio solvent resonances. Elemental analysis was performed externally by Elemental Microanalysis Ltd., Okehampton, Devon, UK. In every case, at least three attempts were made to acquire satisfactory elemental analysis results (i.e., within ±0.5% of the expected C, H and N content). Deuterated solvents (*d*_8_-toluene, C_6_D_6_, *d*_8_-THF) were purchased from Sigma-Aldrich Ltd., dried over a potassium mirror before vacuum distilling under argon and storing over 4 Å molecular sieves. Organic nitriles were used without further purification. Compound **III** was synthesized according to literature conditions.^3a^

#### Synthesis of [K{CH_2_SiMe_2_N(Dipp)}_2_Al(*o*-CH_3_C_6_H_5_CN)_2_]•C_6_D_6_ (1)

In a J Young’s NMR tube, compound **III** (20.0 mg, 0.036 mmol) was dissolved in C_6_D_6_ (ca. 0.5 mL) and *o*-tolunitrile (8.36 mg, 0.071 mmol) was added to the bright yellow *d*_6_-benzene solution. Upon addition of the nitrile, the solution immediately turned golden brown and was kept at room temperature overnight. The, now amber, solution was reduced to half volume *in vacuo* and after 3 h at room temperature, yellow block crystals of **1** (17.2 mg, 61%) suitable for single crystal X-ray diffraction analysis were observed to form. ^1^H NMR (500 MHz, 298 K, *d*_8_-THF, δ): 6.83 (d_app_, 4H, m*-*C_6_H_3_), 6.72–6.69 (m, 4H, Ar*–*H), 6.66–6.62 (m, 2H, *p-*C_6_H_3_), 6.37–6.34 (m, 2H, Ar–H), 6.15–6.13 (m, 2H, Ar–H), 4.14 (s, br, 4H, CHMe_2_), 1.31 (s, 6H, C_6_H_5_Me), 1.24–1.18 (m, 12H, CHMe_2_), 1.16–1.12 (m, 12H, CHMe_2_), 1.07 (s, 4H, SiCH_2_), 0.06 (s, br, 12H, SiMe_2_) ppm. ^13^C{^1^H} NMR (126 MHz, 298 K, *d*_8_-THF) δ 169.3 (PhC=N), 150.3, 147.5 (*i-*C_6_H_4_), 146.3, 137.8 (*i-*C_6_H_3_), 129.9, 129.5, 125.4, 123.6, 123.4, 121.7 (*C*_sp^2^_), 27.9 (CHMe_2_), 26.6, 26.36, 26.0, 20.5 (CHMe_2_), 20.3 (PhMe), 16.3 (SiCH_2_), 1.8 (SiMe_2_) ppm. Despite repeated attempts, a satisfactory CHN microanalysis could not be obtained for this compound.

#### Synthesis of [K{CH_2_SiMe_2_N(Dipp)}_2_Al(*m*-CH_3_C_6_H_5_CN)_2_].2THF (2)

In a J Young’s NMR tube, compound **III** (20.0 mg, 0.036 mmol) was dissolved in C_6_D_6_ (ca. 0.5 mL), and *m*-tolunitrile (8.36 mg, 0.071 mmol) was added to the bright yellow *d*_6_-benzene solution. Upon addition of the nitrile, the solution immediately turned golden brown and was kept at room temperature overnight. The volatiles were removed *in vacuo* and the resulting pale-yellow waxy solid was redissolved in THF. Slow diffusion of *n*-hexane into the THF solution facilitated the deposition of yellow block crystals of **2** (14.8 mg, 52%) suitable for single crystal X-ray diffraction analysis. ^1^H NMR (500 MHz, 298 K, C_6_D_6_, δ): 7.10 (s, 2H, *o-*C_6_H_3_), 6.88–6.87 (m, 4H, Ar–H), 6.85–6.82 (m, 2H, Ar*–*H), 6.78–6.68 (m, 6H, Ar–H), 4.37 (s, br, 4H, CHMe_2_), 2.01 (s, 6H, PhMe), 1.44–1.39 (m, 24H, CHMe_2_), 1.28–1.19 (m, 4H, SiCH_2_), 0.49 (s, br, 12H, SiMe_2_) ppm. ^13^C{^1^H} NMR (126 MHz, 298 K, C_6_D_6_) δ 169.5 (PhC=N), 150.2, 148.0 (*i-*C_6_H_4_), 144.0, 139.4 (*i-*C_6_H_3_), 136.9, 133.8, 132.5 (C_sp_^2^), 129.2, 129.1 (*o-*C_6_H_3_), 127.4, 125.5, 123.1, 121.6, 119.3 (C_sp_^2^), 27.5 (CHMe_2_), 26.1, 25.8, 21.4, 20.7 (CHMe_2_), 15.5 (SiCH_2_), 1.5 (SiMe_2_) ppm. ^13^C resonance correlated to AlC was not observed. Despite repeated attempts, a satisfactory CHN microanalysis could not be obtained for this compound.

#### Synthesis of [K{CH_2_SiMe_2_N(Dipp)}_2_Al-η^2^-NCt-Bu] (3)

In a J Young’s NMR tube, compound **III** (40.0 mg, 0.071 mmol) was dissolved in C_6_D_6_ (ca. 0.5 mL), after which *t*-BuCN (5.93 mg, 0.071 mmol) was added. The bright yellow solution was kept at room temperature for 3 days, after which a crop of yellow block crystals of compound **3** (26.9 mg, 59%) was observed to deposit. ^1^H NMR (500 MHz, 298 K, *d*_8_-THF, δ): 6.93 (d_app_, 4H, Ar–H), 6.81–6.78 (m, 2H, Ar*–*H), 4.10 (s, br, 2H, CHMe_2_), 3.80–3.69 (m, 2H, CHMe_2_), 1.29 (m, 12H, CHMe_2_), 1.21–1.13 (m, 12H, CHMe_2_), 0.95 (s, 4H, SiCH_2_), 0.30 (s, 9H, *t*-*Bu* CH_3_), −0.04 (s, br, 12H, SiMe_2_) ppm. ^13^C{^1^H} NMR (126 MHz, 298 K, *d*_8_-THF, δ): 150.6 (C_sp_^2^), 150.4 (C_sp_^2^), 150.3 (C_sp_^2^), 147.7 (C_sp_^2^), 147.5 (C_sp_^2^), 123.9 (C_sp_^2^), 123.5 (C_sp_^2^), 122.8 (C_sp_^2^), 122.2 (C_sp_^2^), 120.8 (C_sp_^2^), 41.1, 31.9, 28.4, 28.0, 27.2, 26.7, 24.3 (CHMe_2_), 15.5 (SiCH_2_), 1.3 (SiMe_2_) ppm. Despite repeated attempts, a satisfactory CHN microanalysis could not be obtained for this compound.

#### Synthesis of [K{CH_2_SiMe_2_N(Dipp)}_2_Al-κ^*N*,*N*′^ -{(H)N(i-Pr)C=C(C_3_H_5_)N(H)}].THF (4)

In a J Young’s NMR tube, compound **III** (40.0 mg, 0.071 mmol) was dissolved in C_6_D_6_ (ca. 0.5 mL), after which *i*-PrCN (9.87 mg, 0.143 mmol) was added. The bright yellow solution was then kept at room temperature overnight. The volatiles were removed *in vacuo* and the resulting off-white solid was redissolved in a mixture of *n*-hexane and THF. Colorless block crystals of **4** (13.1 mg, 51%) deposited at room temperature. ^1^H NMR (500 MHz, 298 K, *d*_8_-THF, δ): 6.95–6.84 (m, 6H, Ar*–*H), 4.11 (s, br, 4H, Dipp–CHMe_2_), 3.86 (br. S, 1H, C=CH), 3.81 (br. S, 1H, C=CH), 3.62 (m, 4H, THF CH_2_), 2.83 (hept, ^3^*J*_HH_ = 6.9 Hz, 1H, CHMe_2_), 2.51 (br s, NH), 1.80 (s, 3H,=CMe), 1.77 (m, 4H, THF CH_2_), 1.25–1.11 (m, 32H, CH_3_), 0.92 (s, br, 4H, SiCH_2_) 0.35 (s, br, 6H, SiMe_2_), −0.47 (s, br, 6H, SiMe_2_) ppm. ^13^C{^1^H} NMR (126 MHz, 298 K, *d*_8_-THF, δ): 145.5 (*i-*C_6_H_3_), 142.1 (*o-*C_6_H_3_), 122.0 (*m-*C_6_H_3_), 118.0 (*p-*C_6_H_3_), 97.3 (AlNC), 68.4 (C=CMe_2_), 29.2, 28.3 (CHMe_2_), 26.6, 24.8, 22.4 (CHMe_2_), 15.9 (SiCH_2_), 1.9 (SiMe_2_) ppm. Despite repeated attempts, a satisfactory CHN microanalysis could not be obtained for this compound.

## Results and Discussion

### Reactivity of III with Aryl Nitriles

**Scheme 2:**
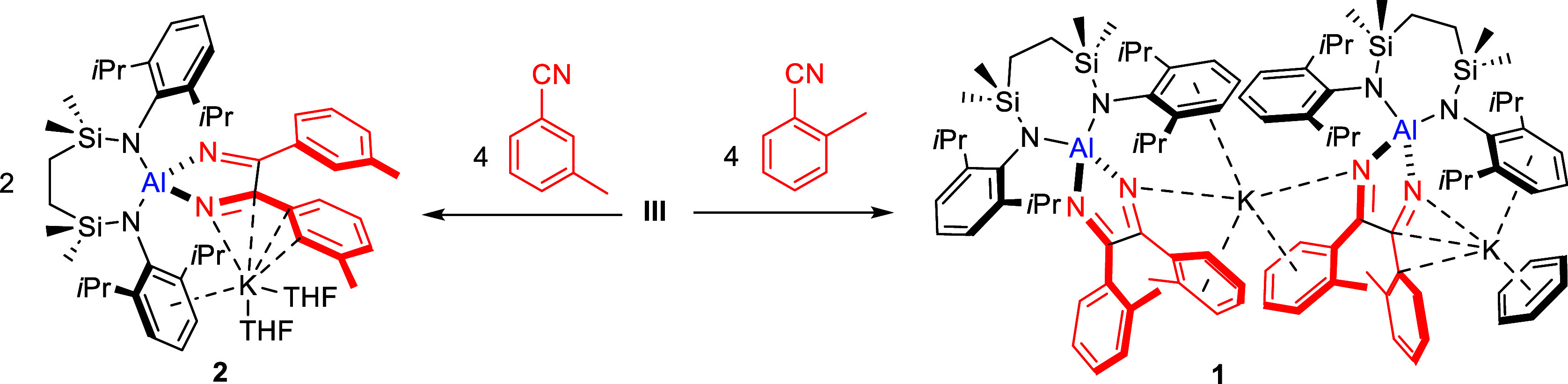
Synthesis of Compounds **1** and **2**

Informed by our previous study of magnesium boryl reactivity toward nitriles,^27^*o*- and *m*-tolunitrile were selected as *C-*aryl nitrile substrates for reaction with compound **III** due to their potential to provide straightforward diagnostic evaluation by monitoring of the aromatic methyl substituent by NMR spectroscopy. Although, in both cases, a new product was observed to form by ^1^H NMR spectroscopy after addition of a stoichiometric equivalent of each aryl nitrile to **III**, ca. 50% of the alumanyl reagent was observed to remain unreacted. Adjustment of the reactions to a 2:1 stoichiometry, however, yielded the exclusive formation of compounds **1** and **2**, for the *o*- and *m*-tolunitrile reactions, respectively (Scheme 2). The complete consumption of **III** was evidenced by the disappearance of its diagnostic Dipp iso-propyl multiplet at δ 3.97 ppm. In each case, this occurred with the concurrent emergence of a lower field multiplet in a similar chemical shift region (δ 4.22 and 4.37 ppm for **1** and **2**, respectively). Sharp 6H singlet resonances assigned as the *o*- and *m*-methyl substituents at δ 1.31 (**1**) and 2.01 (**2**) ppm suggested the incorporation of two C–C conjoined nitrile units with a symmetrical *N*,*N*′-chelated disposition of the two former nitrile moieties about aluminum. This outcome is strongly reminiscent of Roesky’s synthesis of **XI** (Scheme 1a),^23^ our own observations of the identical nitrile substrates toward magnesium boryl equivalents,^27^ and the 2 electron C–C coupling process observed by Jones and co-workers upon addition of benzonitrile to reducing β-diketiminato Mg(I) dimers.^22a^

**Figure 2:**
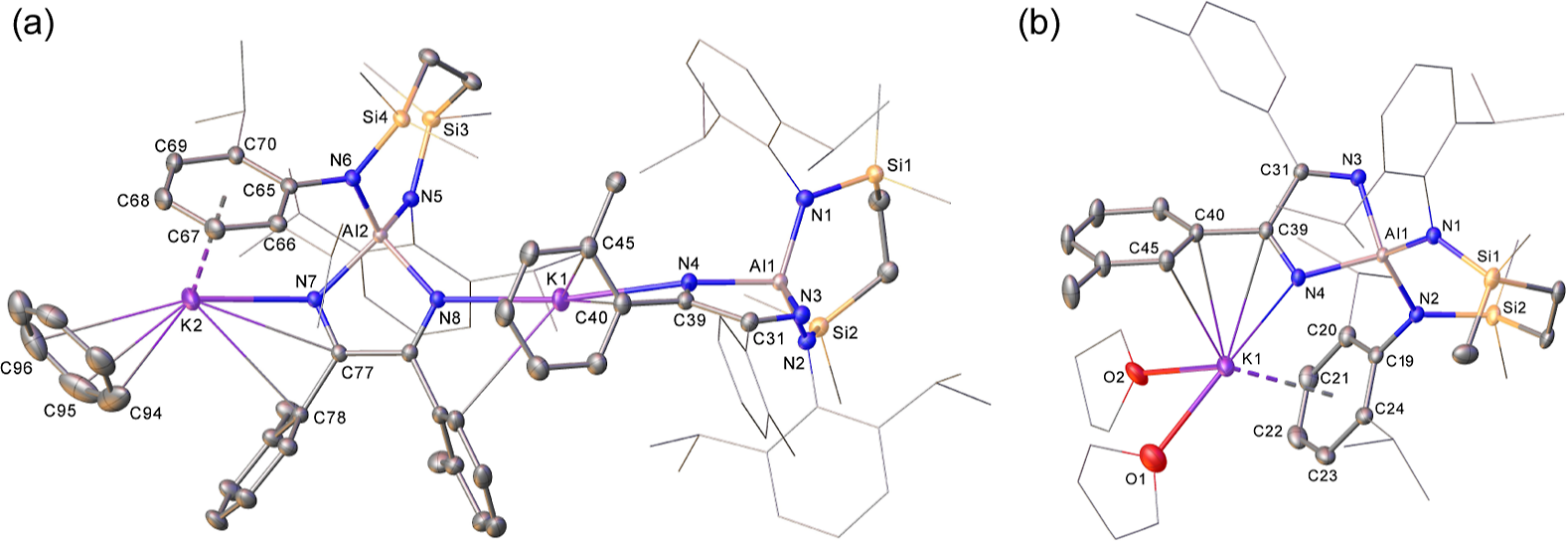
(a) Plot depicting the molecular structure of compound **1**. Ellipsoids are depicted at 30% probability. Hydrogen atoms have been omitted and peripheral substituents are depicted as wireframes, for visual ease. (b) Plot depicting the molecular structure of compound **2**. Ellipsoids are depicted at 30% probability. Solvent, the minor disordered component atoms and hydrogen atoms have been omitted for clarity while peripheral substituents are depicted as wireframes, also for visual ease.

These deductions were confirmed by X-ray diffraction analysis of **1** and **2**, performed on single crystals obtained from benzene and THF solutions, respectively. The aluminum centers in both compounds present pseudo-tetrahedral coordination geometries provided by a combination of the chelated {SiN^Dipp^} ligands and the relevant C-*ortho* and C-*meta*-tolyl diazabutadiene dianions (Figure 2, Table 1), such that the aluminum centers comprise what are now tetra-aza-aluminate units. The major point of distinction between the two compounds in the solid state is provided by the molecularity of the structures, which may be attributed to the differing conditions of their crystallization. Compound **1** is isolated from benzene as a dimeric structure in which the Al1- and Al2-containing aluminate moieties are connected by interactions of K1 with the nitrogen donors [K1–N4 2.8419(16), K1–N8 2.8454(15) Å] provided by each diazabutadiene ligand. The encapsulation of K1 is completed through polyhapto engagement by the proximal [C40- and C86-containing] *o*-tolyl substituents. Although K2 interacts through similar aryl interactions with the remaining *o*-tolyl group and a Dipp substituent of the Al2-containing aluminate, further intermolecular aggregation of **1** is prevented by an additional multihapto–arene interaction of this potassium with a capping molecule of benzene. Any potential for similar oligomerization of **2** is apparently prevented by the presence of O-donor solvent. Although K1 again binds to the aluminate component of **2** through a combination of polyhapto interactions with the Dipp and *m-*tolyl substituents, this alkali metal cation is also bound by two molecules of THF, which impose a monomeric, contact ion paired structure. Despite these differences, the analogous bond lengths and angles across the two structures (Table 1) are broadly comparable. In both cases, the Al–N bond lengths to the {SiN^Dipp^} ligands [**1**: Al1–N1 1.8570(17), Al1–N2 1.8564(17), Al2–N5 1.8537(16), Al2–N6 1.8657(17); **2**: Al1–N2 1.8574(12), Al1–N1 1.8526(13) Å] are very similar and are commensurate with previously observed bond lengths observed after the formal two electron oxidation of **III**.^3^ In comparison to these interactions, the various Al–N bonds to the diazabutadiene dianions in both structures are elongated [ca. 1.91–1.92 Å], while the C–N (ca. 1.26 Å, irrespective of any addition engagement to potassium) and C–C bonds [**1**: C31–C39 1.572(3), C77–C85 1.569(2); **2**: C31–C39 1.570(2) Å] resulting from the coupling of the aryl nitrile reagents, best identify each {N=(Ar)C–C(Ar)=N}^2–^ unit as comprising alternating and effectively localized C=N double and C–C single bonds.

**Table 1:** Selected Bond Distances (Å) and Bond Angles (°) for Compounds **1** and **2**

	**1**	**2**
Al1–N1	1.8570(17)^a^	1.8526(13)
Al1–N2	1.8564(17)^b^	1.8574(12)
Al1–N3	1.9141(17)^c^	1.8966(13)
Al1–N4	1.9397(16)^d^	1.9224(12)
N3–C31	1.260(3)^e^	1.2652(19)
N4–C39	1.265(3)^f^	1.2746(19)
C31–C39	1.572(3)^g^	1.570(2)
N1–Al1–N2	112.35(8)^h^	112.40(6)
N3–Al1–N4	90.94(7)^i^	91.80(6)

a Al2–N5 1.8537(16) Å.

b Al2–N6 1.8657(17) Å.

c Al2–N7 1.9177(16) Å.

d Al2–N8 1.9293(16) Å.

e N7–C77 1.267(3) Å.

f N8–C85 1.260(3) Å.

g C77–C85 1.569(2) Å.

h N5–Al2–N6 116.21(7)°.

i N7–Al2–N8 90.72(7).

### Reactivity of III with Alkyl Nitriles

**Scheme 3:**
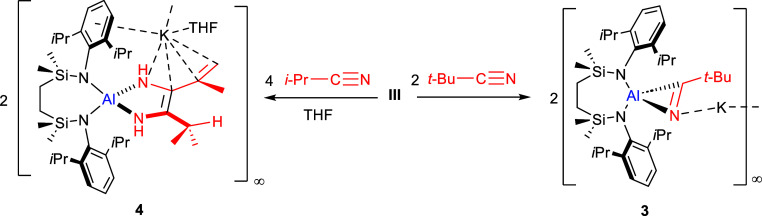
Synthesis of Compounds **3** and **4**

Prompted by Roesky’s observation of the formation of compound **XI** and the proposed intermediacy of compound **X** (Scheme 1a),^23^ compound **III** was reacted with *t*-BuCN. In contrast to the 2:1 nitrile to alumanyl stoichiometry required for the synthesis of compounds **1** and **2**, monitoring of a reaction of a stoichiometric equivalent of *t*-BuCN with **III** in C_6_D_6_ by ^1^H NMR spectroscopy evidenced a single new species (**3**, Scheme 3), characterized by the emergence of a broad Dipp *i*-Pr signal at δ 4.10 ppm. The appearance of this latter resonance coincided with that of an intense (9H) singlet at δ 0.63 ppm, representing a significant upfield shift in comparison to the methyl resonance arising from free *t*-BuCN (δ 1.02 ppm).

**Figure 3:**
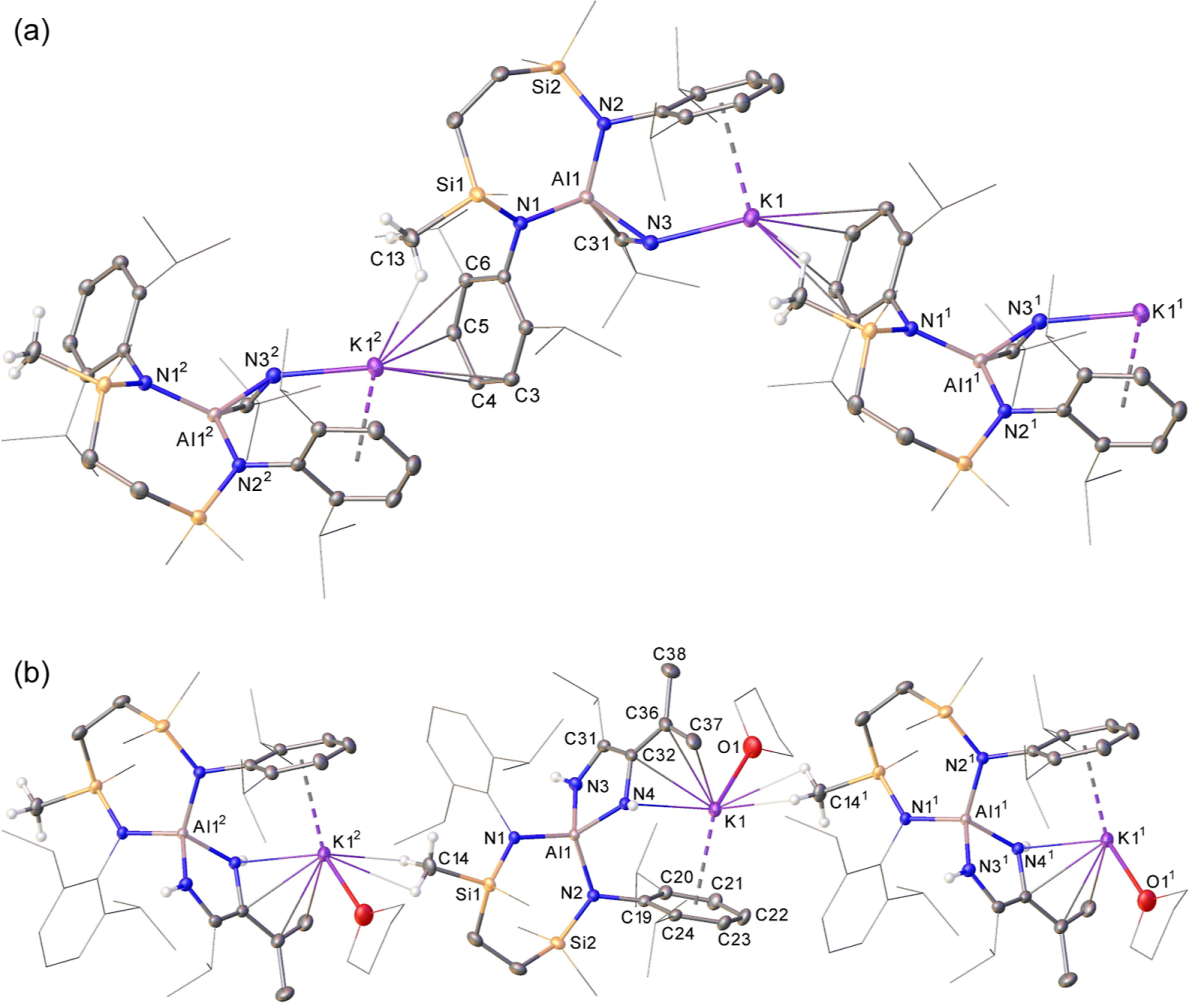
(a) Plot depicting the molecular structure of **3**. Ellipsoids are depicted at 30% probability. Hydrogen atoms have been omitted (C13 excepted) and peripheral substituents are depicted as wireframes, for clarity. Symmetry operations:^1^ 1/2 – *x*, −1/2 + *y*, *z*;^2^ 1/2 – *x*, 1/2 + *y*, *z*. (b) Plot depicting the molecular structure of **4**. Ellipsoids are depicted at 30% probability. The minor disordered component atoms and hydrogen atoms (those attached to N3, N4 and C14 excepted) have been omitted for clarity while peripheral substituents are depicted as wireframes, also for visual perspicuity. Symmetry operations:^1^ 1 – *x*, −1/2 + *y*, 1/2 – *z*;^2^ 1 – *x*, 1/2 + *y*, 1/2 – *z*.

After 3 days at room temperature, a crop of yellow block crystals was observed to deposit from the reaction solution. Compound **3** was confirmed by single crystal X-ray diffraction analysis to be a [2 + 1] cycloaddition product, which, like the intermediate **X** proposed by Roesky and co-workers,^23^ consists of a three-membered heterocycle with η^2^-*N*,*C*-coordination toward the aluminum center provided by the reduced nitrile moiety (Figure 3a, Table 2). Examples of analogous η^2^-nitrile coordination have previously been reported for a variety of transition metals.^28^ Literature precedent for a similar η^2^-mode of nitrile engagement to any *p-*block group element is, however, and to the best of our knowledge, limited to three examples of *C-*diorgano-2H-azirines, [(2,4,6-{CH(SiMe_3_)_2_}_3_C_6_H_2_)(2,4,6-Me_3_C_6_H_2_)Si(η^2^-NC*t-*Bu)] and [{H_2_C(SiMe_3_)_2_}_2_Si(η^2^-NC-4-X-C_6_H_4_)] (X=Cl, OMe) that resulted from treatment of nitriles with the relevant diorganosilylenes.^29^ Although we are now continuing to study the further reactivity of compound **3**, its isolation lends further credence to Roesky’s proposal that compound **XI** does indeed result from a stepwise [2 + 1] insertive process (Scheme 1).^23^

**Table 2:** Selected Bond Distances (Å) and Bond Angles (°) Compounds **3** and **4**

	**3**	**4**
Al1–N1	1.8576(12)	1.8674(11)
Al1–N2	1.8530(12)	1.8710(11)
Al1–N3	1.8876(13)	1.8506(12)
Al1–N4		1.8570(12)
Al1–C31	1.9025(14)	
N3–C31	1.298(2)	1.3809(18)
N4–C32		1.4354(17)
C31–C32		1.385(2)
C32–C36		1.461(2)
C36–C37		1.343(3)
C36–C38		1.521(2)
N4–C32		1.4354(17)
N3–K1	2.6138(13)	2.8026(12)^a^
N1–Al1–N2	113.24(5)	111.83(5)
N1–Al1–N3	123.81(5)	110.27(5)
N1–Al1–C31	120.21(6)	86.05(5)^b^
Al1–N3–C31	70.59(8)	112.74(9)

a N4–K1.

b N3–Al1–N4.

Evidence for two-electron reduction of the nitrile moiety in **3** is clearly documented through comparison of the C=N bond distance [N(3)–C(31) = 1.298(2) Å] with that of a C≡N triple bond in which *t*-BuCN acts as a conventional κ^1^-*N*-coordinated ligand to a purely σ-acidic main group element center [1.137(5) Å].^30^ In further support of this conclusion, and consistent with formal Al(I) to Al(III) oxidation, the Al–N bond lengths of the [{SiN^Dipp^}Al] unit [Al1–N1 1.8530(12), Al1–N2 1.8576(12) Å] are again shorter than those of **III** [Al1–N1 1.887(2), Al1–N2 1.8889(19) Å], while the N–Al–N bond angle is significantly more obtuse [**3**: 113.24(5)°; **III** 108.84(9)°] despite the seven-membered chelate structure.^3^ As depicted in Figure 3a, compound **3** exhibits a polymeric structure in the solid state propagated by intermolecular K1–N3 [2.6138(13) Å] and polyhapto interactions between the potassium cations and aromatic Dipp substituents of adjacent aluminate moieties. The formation of this extended molecular network also rationalizes the spontaneous crystallization of **3** from the C_6_D_6_ reaction mixture at room temperature and dictated that its complete solution state characterization was necessarily conducted in *d*_8_-THF.

To further assess the impact of incremental reduction in the steric demands of the nitrile reagent, a stoichiometric reaction of *i*-PrCN with **III** was performed. Although monitoring by ^1^H NMR spectroscopy indicated the formation of a new {SiN^Dipp^}-supported species through the emergence of an *i*-Pr multiplet at δ 4.13 ppm, a large quantity of unreacted potassium alumanyl continued to be identified, even after prolonged reaction times and heating to 60 °C. As was the case in the synthesis of compounds **1** and **2**, complete consumption of **III** and the generation of a single new compound (**4**) could only be achieved through an increase of the reaction stoichiometry to a 2:1 ratio of the nitrile and alumanyl reagents (Scheme 3). Consistent with the generation of a more asymmetric aluminum center, the ^1^H NMR resonance associated with the [{SiN^Dipp^}] ligand protons of compound **4** was broad. Although attempted crystallization from the reaction solution was unsuccessful, a mixture of *n*-hexane and THF yielded colorless single block crystals of compound **4** allowing a resolution of the solution state observations by single crystal X-ray diffraction analysis (Figure 3b).

Like **1** and **2**, compound **4** presents a spirocyclic tetra-aza-aluminate anion derived from the C–C coupling of two nitrile molecules at the Al(I) center of **III**. In this instance, however, consideration of the N3–C31 [1.3809(18) Å], C31–C32 [1.385(2) Å], C32–N4 [1.4354(17) Å], C32–C36 [1.461(2) Å] and C36–C37 [1.343(3) Å] bond lengths across the resultant heterocycle indicate that it is best now viewed as an alumina-*N*,*N*′-1,2-diaminobutadiene moiety (Table 2). In addition, hydrogen atoms were located and refined at a distance of 0.96 Å from both N3 and N4, indicating the operation of an unusual isomerization process requiring the migration of both the methine hydrogen and a single methyl hydrogen of a nitrile iso-propyl substituent. While there are no grounds at present to attempt a more definitive interpretation of this outcome, the balance of probability advocates the C–C coupling process again occurs through initial [2 + 1] nitrile addition of to the Al(I) center of **III**. Irrespective of the subsequent order of events, it appears likely that the observed isomerization is sterically induced by the excessive crowding of the [{SiN^Dipp^}Al] imposed by the ensuing reaction of the *C*-*iso*-propyl analogue of compound **3** with a further equivalent of *i*-PrCN. Despite the presence of a coordinated molecule of THF, compound **4** again presents a one-dimensional polymer in the solid state, arising from a combination of intramolecular π-interactions to potassium by both the resultant *N*,*N′*-1,2-diaminobutadiene linkage and a {SiN^Dipp^} aryl substituent [K···C range = 2.8716(14)-3.2180(13) Å] and close intermolecular contacts to the protons attached to C14 of an adjacent aluminate structure. Although this polymeric structure dictated that further analysis by NMR spectroscopy had to be performed in *d*_8_-THF, the resultant ^1^H NMR spectrum provided further corroboration of its structure. The methylene unit could be assigned as two broadened (1H by relative integration) resonances at δ 3.86 and 3.81 ppm, while a sharp heptet of a similar intensity at δ 2.83 ppm was consistent with the presence of only one intact C*H*Me_2_ unit arising from the incorporation of two molecules of *i*-PrCN. A diagnostic resonance in the corresponding ^13^C NMR spectrum at δ 97.3 ppm was strongly reminiscent of the similar enolate environment produced by the C–H addition reaction resulting from treatment of **III** with 2,4-dimethylpentanone,^18a^ and was, thus, assigned as the newly formed tertiary sp^2^ carbon.

## Conclusions

In common with a number of strained alumina-heterocyclic structures provided by the reactivity of a multiply bonded substrates with the neutral derivative [{HC(C(Me)NDipp)_2_}Al] (**XII**),^23,26,31^ and the reactivity of alumanyl reagents, for example **II**, with 1,3,5,7-cyclooctadiene^2^ or ethene,^15b^ the empirical evidence presented herein argues that the initial step in the generation of both compounds **XI** and **1** – **4** occurs via a common mode of [2 + 1] cycloaddition. On this basis, it is suggested that subsequent tolunitrile insertion provides facile access to the alumina diazabutadiene structures of compounds **1** and **2** by what is best considered as a regioselective polarized insertion of the nitrile C≡N bond. The increased steric profile of alkyl nitriles, however, can impose a marked effect on the nature of the products formed. The reaction of **III** with *t*-BuCN provides an alumina cyclopropane unit that is kinetically resistant to onward reaction with a further nitrile equivalent and corroborates the likely operation of a pathway predicated on initial [2 + 1] cycloaddition. Although reduction in the alkyl nitrile steric demands by use of *i*-PrCN again facilitates C–C bond formation, the resultant alumina diazabutadiene divide species appears to be beyond the limit of kinetic viability, inducing an unusual C–H to N–H isomerization at one of the *iso*-propyl substituents. We are continuing to study this chemistry and related reactivity.

## Supporting Information Available

NMR spectra and crystallographic data, details of the computational analysis (PDF)
