# Supplementary material for: Alumanyl Reduction, Reductive Coupling and C–H Isomerization of Organic Nitriles
Source: Organometallics. 2024 Aug 16;43(17):1938–45. doi: 10.1021/acs.organomet.4c00289 (PMC11388447; doi:10.1021/acs.organomet.4c00289)
Supplement: Supplementary file 1 — om4c00289_si_001.pdf [file om4c00289_si_001.pdf]

Supporting Information for:

Alumanyl Reduction, Reductive Coupling and C-H Isomerization of Organic nitriles

Henry T. W. Shere, Han-Ying Liu, Michael S. Hill* and Mary F. Mahon

Department of Chemistry, University of Bath, Claverton Down, Bath, BA2 7AY, UK

Figure S1: ^{1}H NMR (500 MHz, 298 K, d_8-THF) of compound **1**. (**o*-toluCN, # silicone grease)

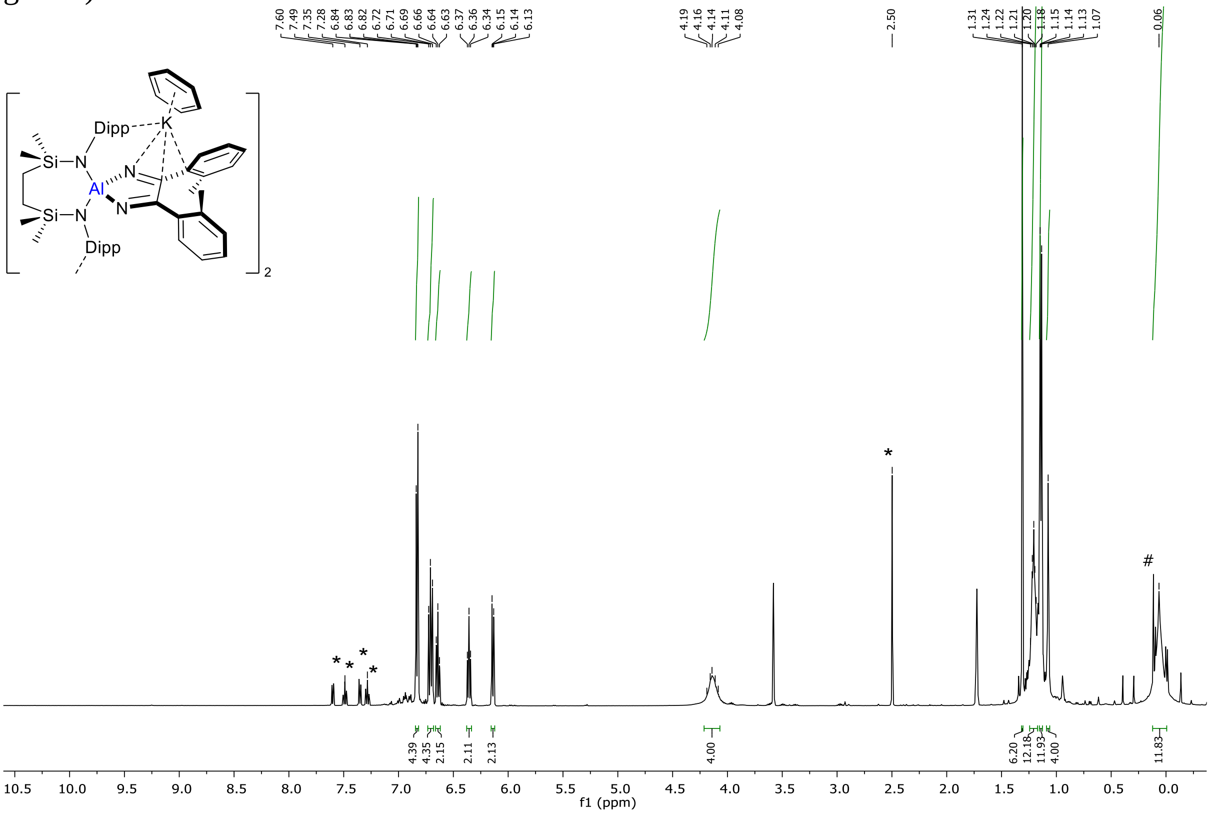

Figure S2: ^{13}C{^{1}H} NMR (126 MHz, 298 K, d_8-THF) of compound **1**. (**o*-toluCN, # silicone grease).

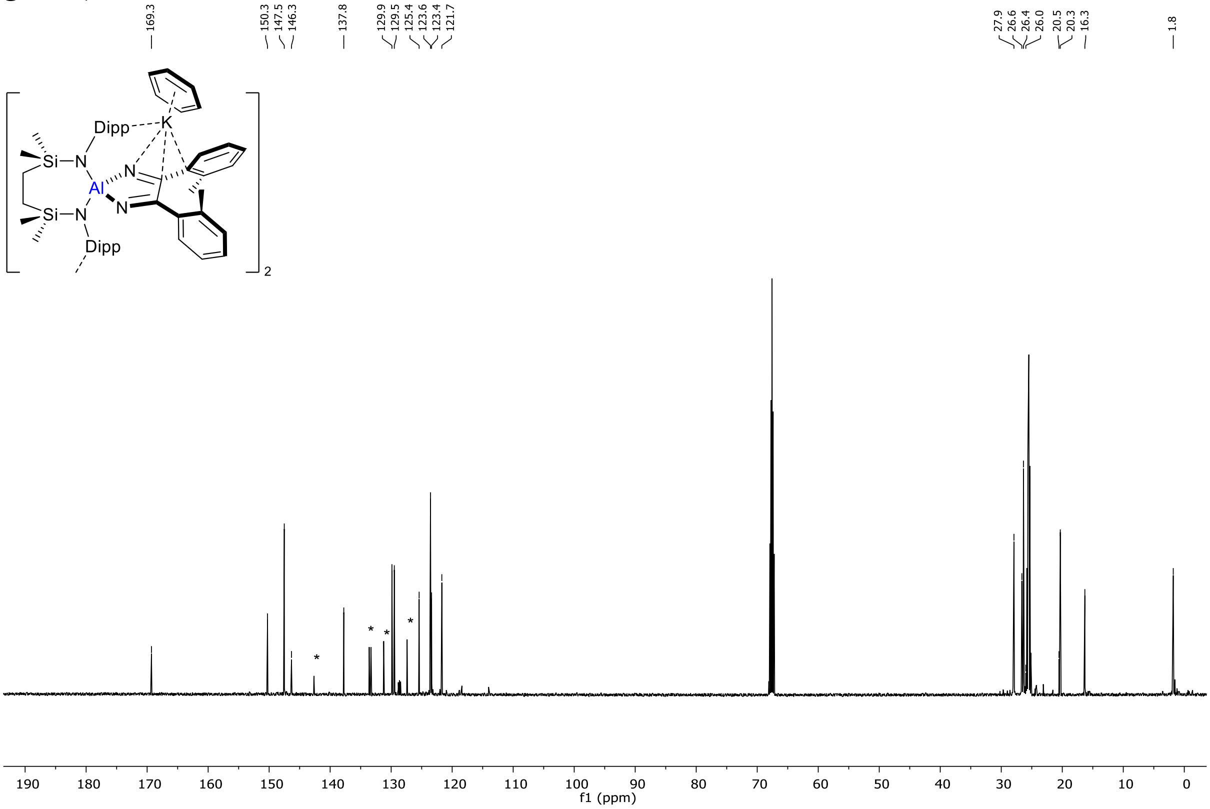

Figure S3: ^{1}H-^{13}C HSQC trace of compound **1**.

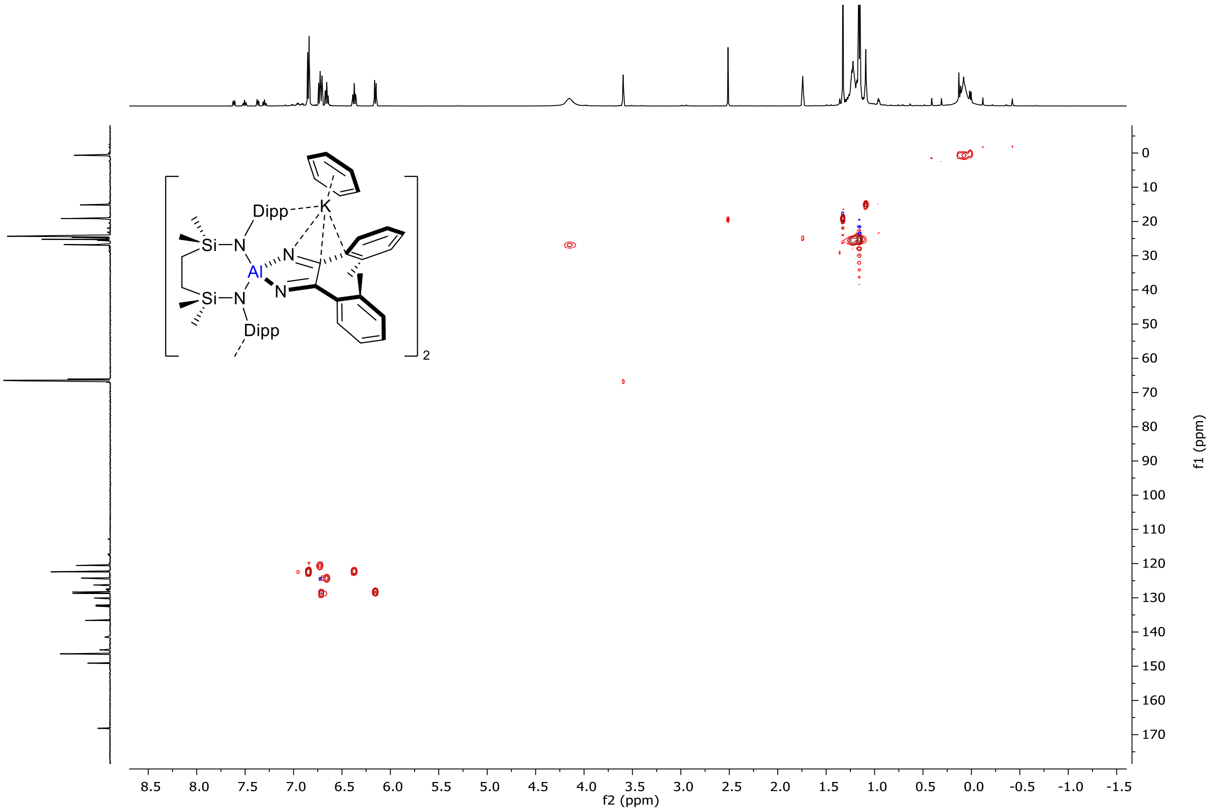

Figure S4: ^{1}H NMR (500 MHz, 298 K, Benzene-*d*6) of compound **2**. (**m*-toluCN, # silicone grease).

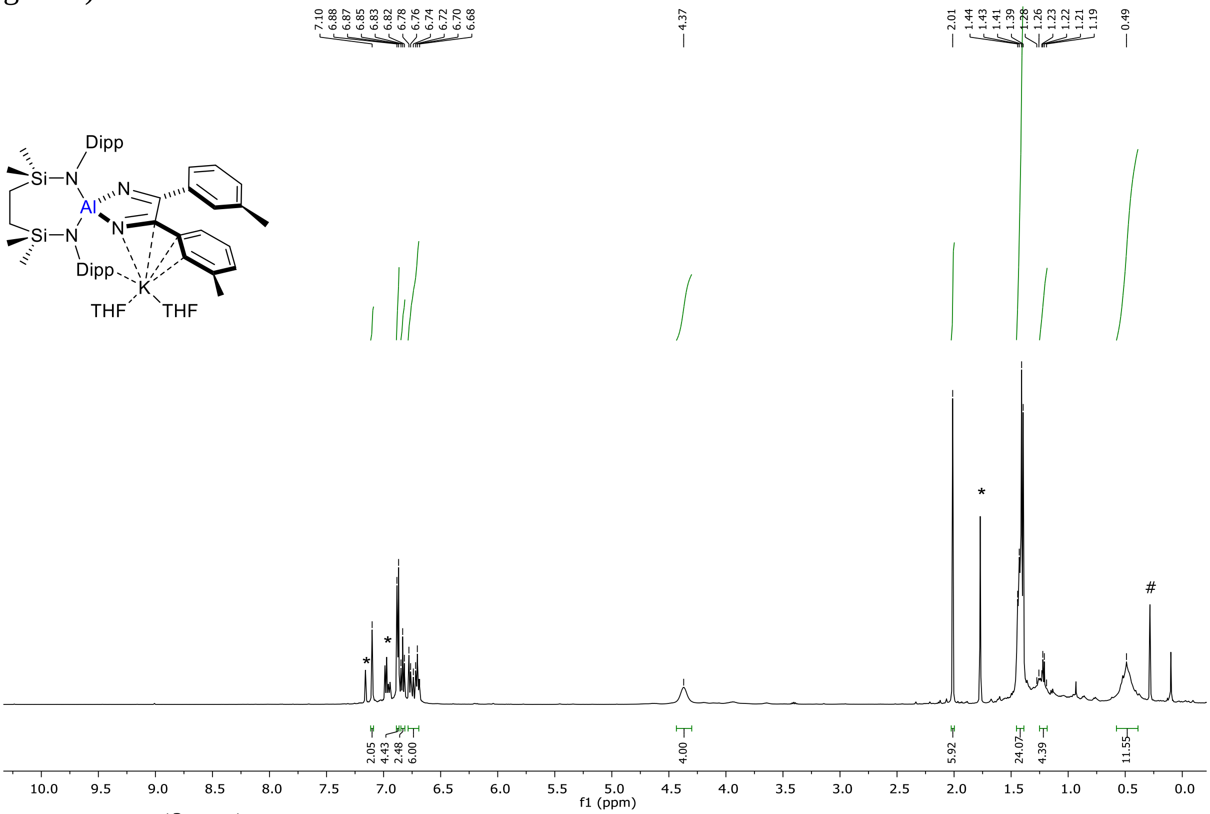

Figure S5: ^{13}C {^{1}H}NMR (126 MHz, 298 K, Benzene-*d*6) of compound **2**. (**m*-toluCN, # silicone grease).

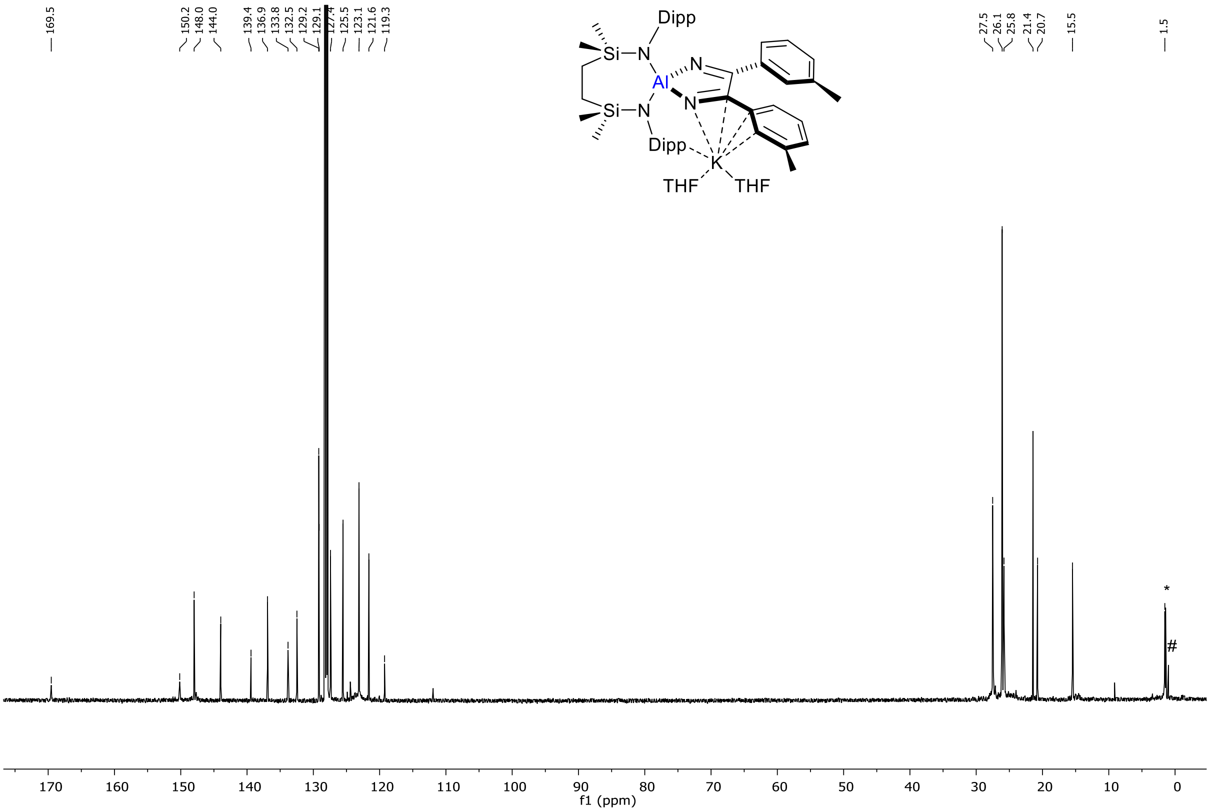

Figure S6: ^{1}H-^{13}C HSQC trace of compound **2**.

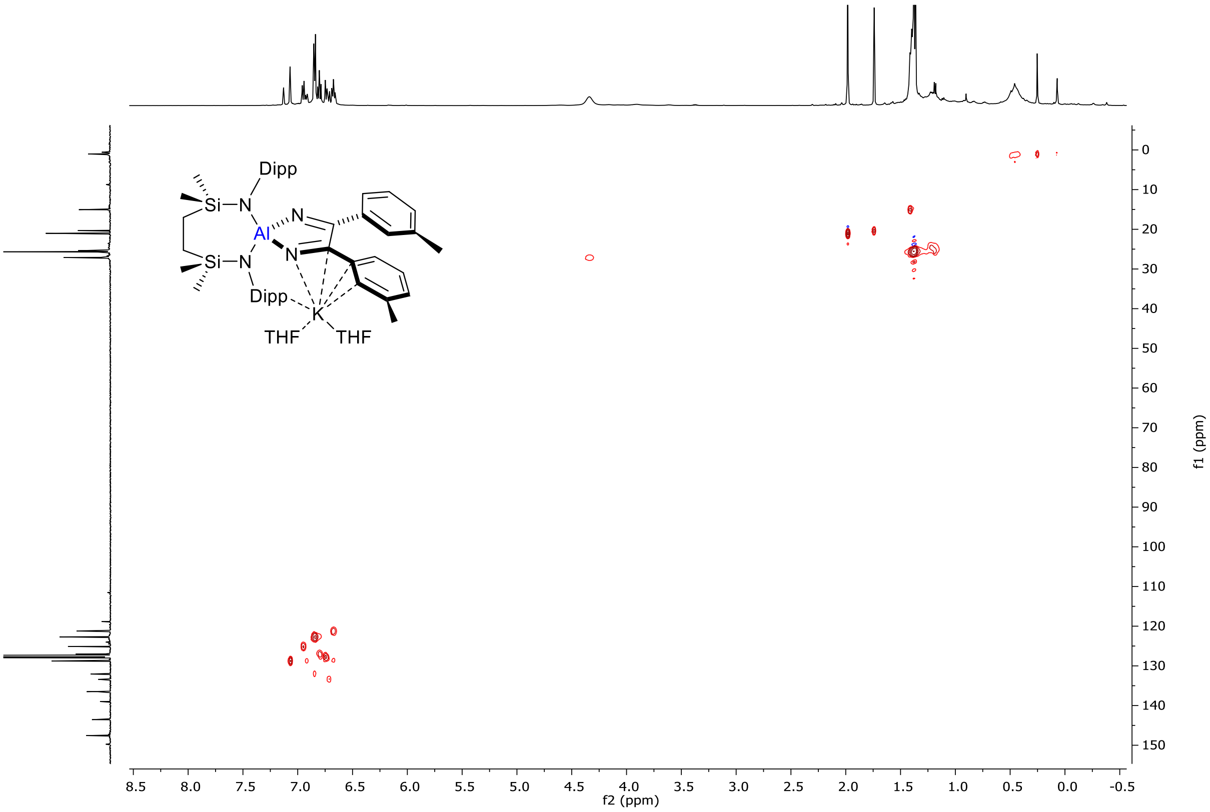

Figure S7: ^{1}H NMR (500 MHz, 298 K, d_8-THF) of compound **3**. (* *t*-BuCN)

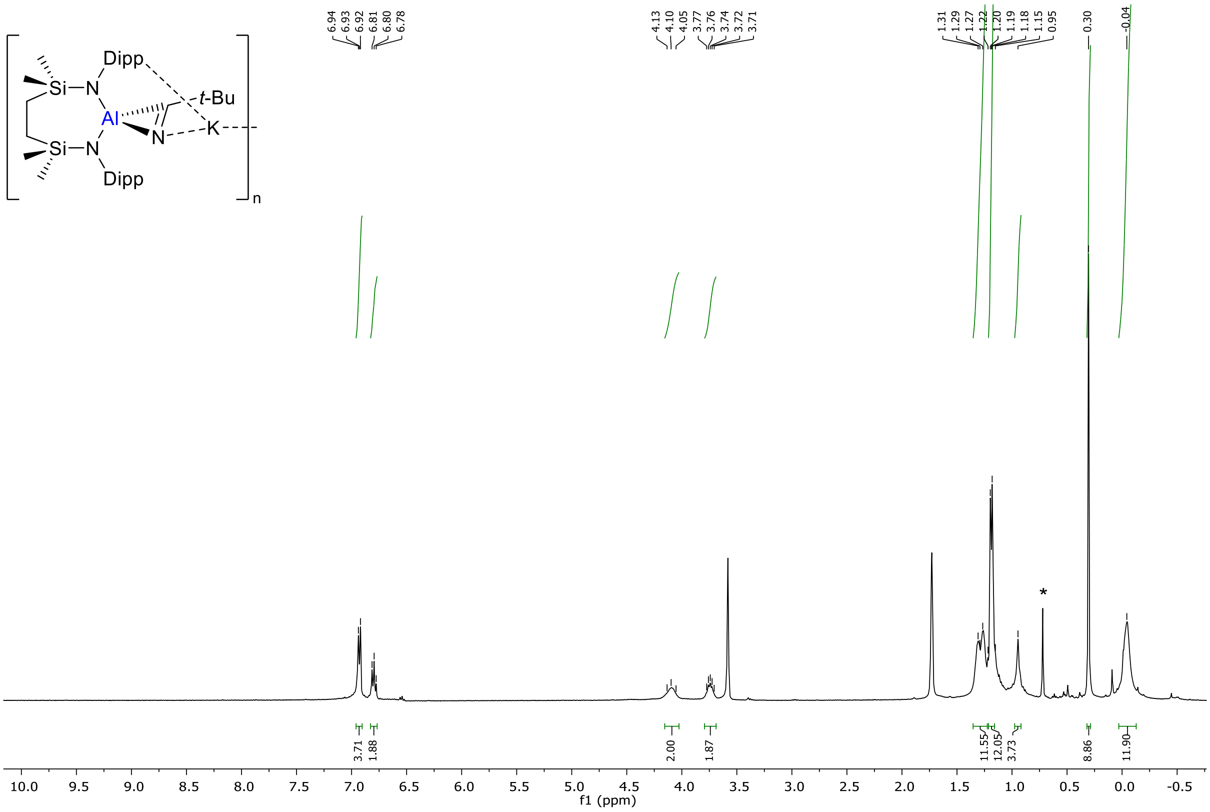

Figure S8: ^{13}C{^{1}H} NMR (126 MHz, 298 K, d_8-THF) of compound **3**. (* *t*-BuCN)

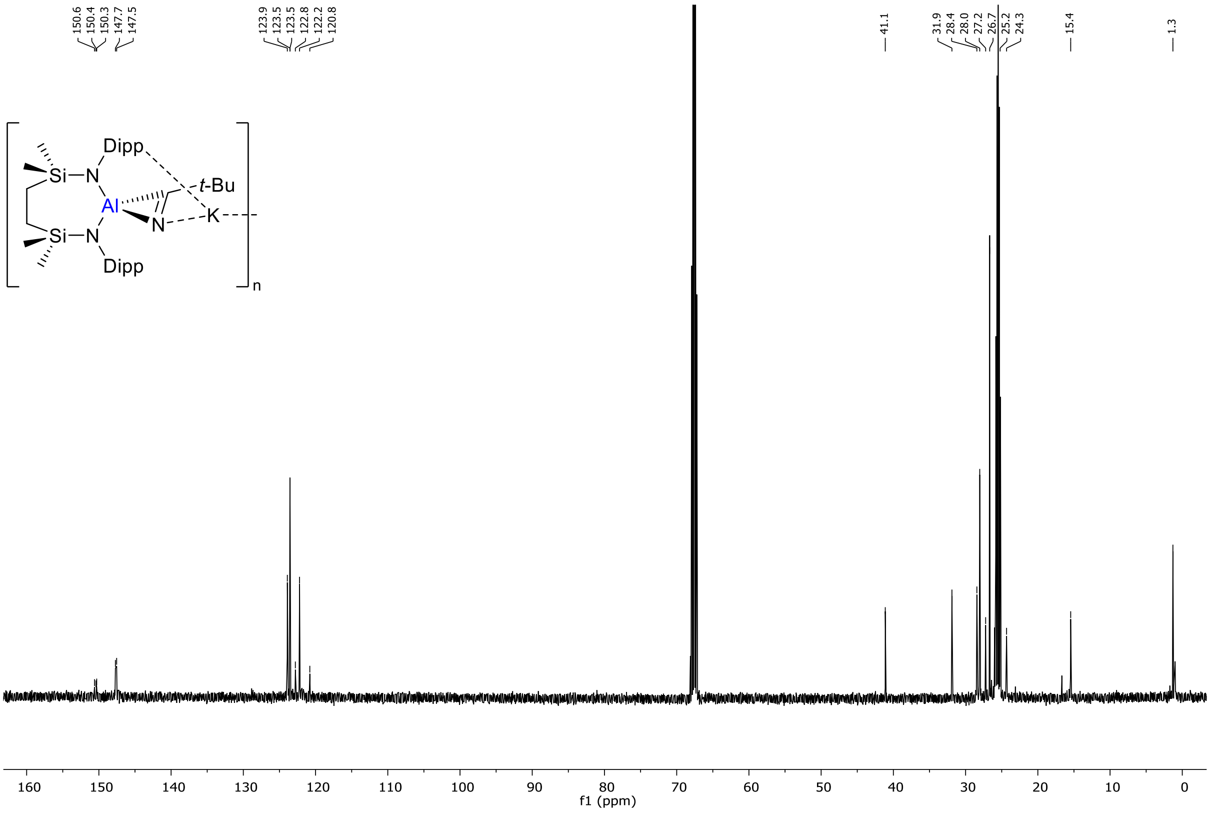

Figure S9: ^{1}H NMR (500 MHz, 298 K, d_8-THF) of compound **4**.

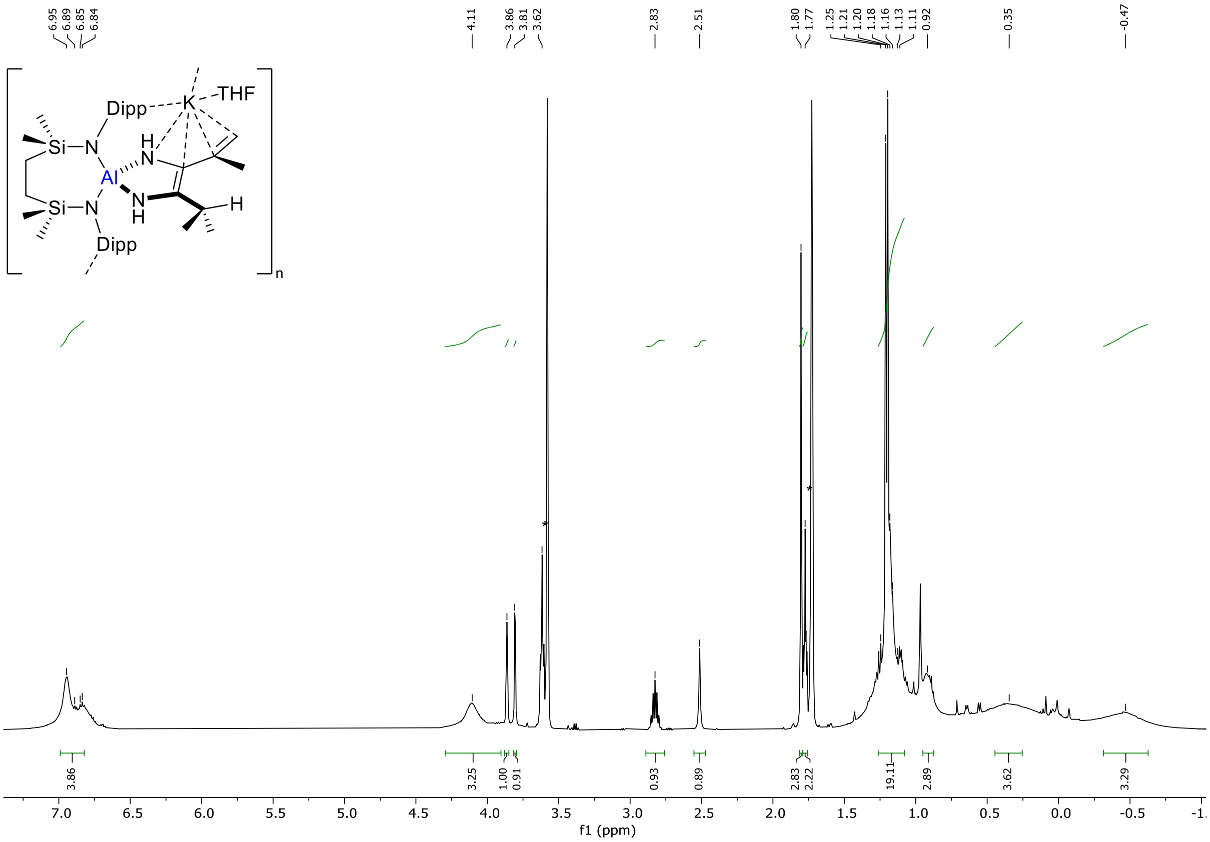

Figure S10: ^{13}C{^{1}H} NMR (126 MHz, 298 K, d_8-THF) of compound **4**.

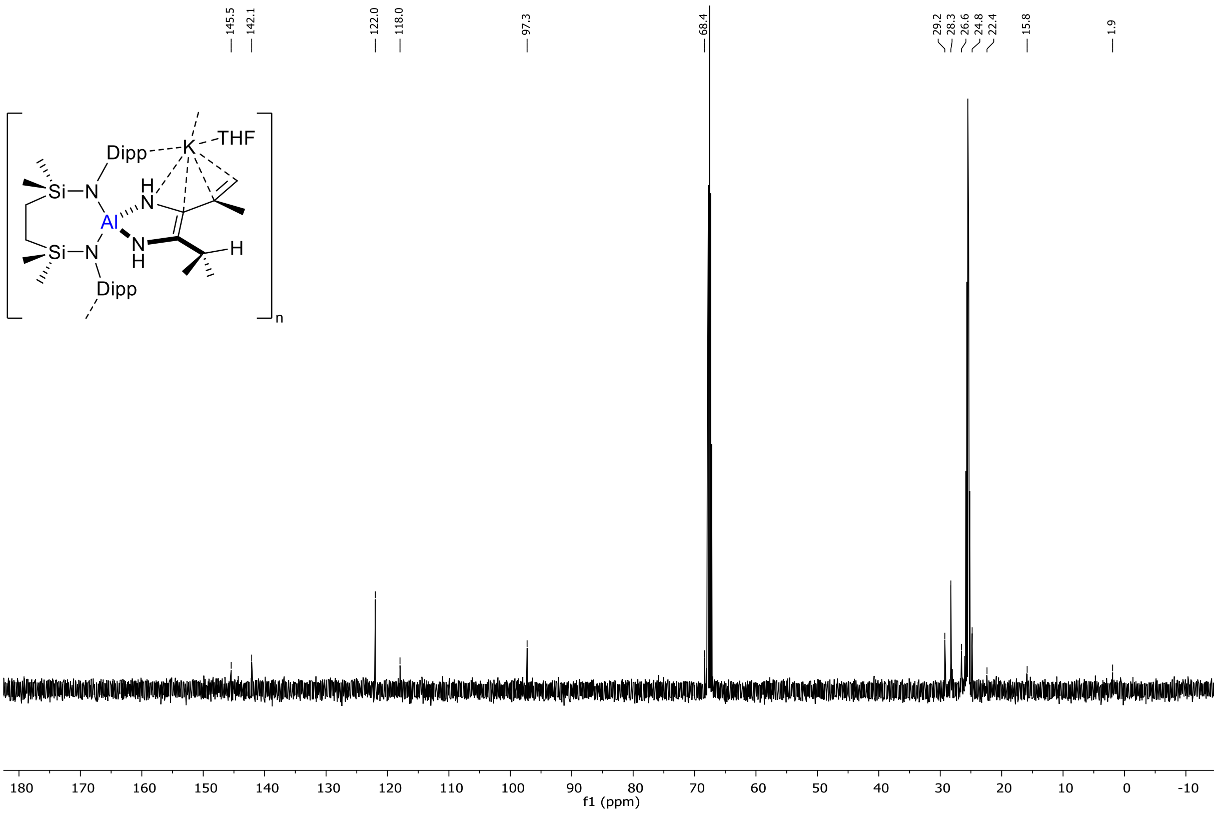

Figure S11: ^{1}H-^{1}H COSY trace of compound **4**.

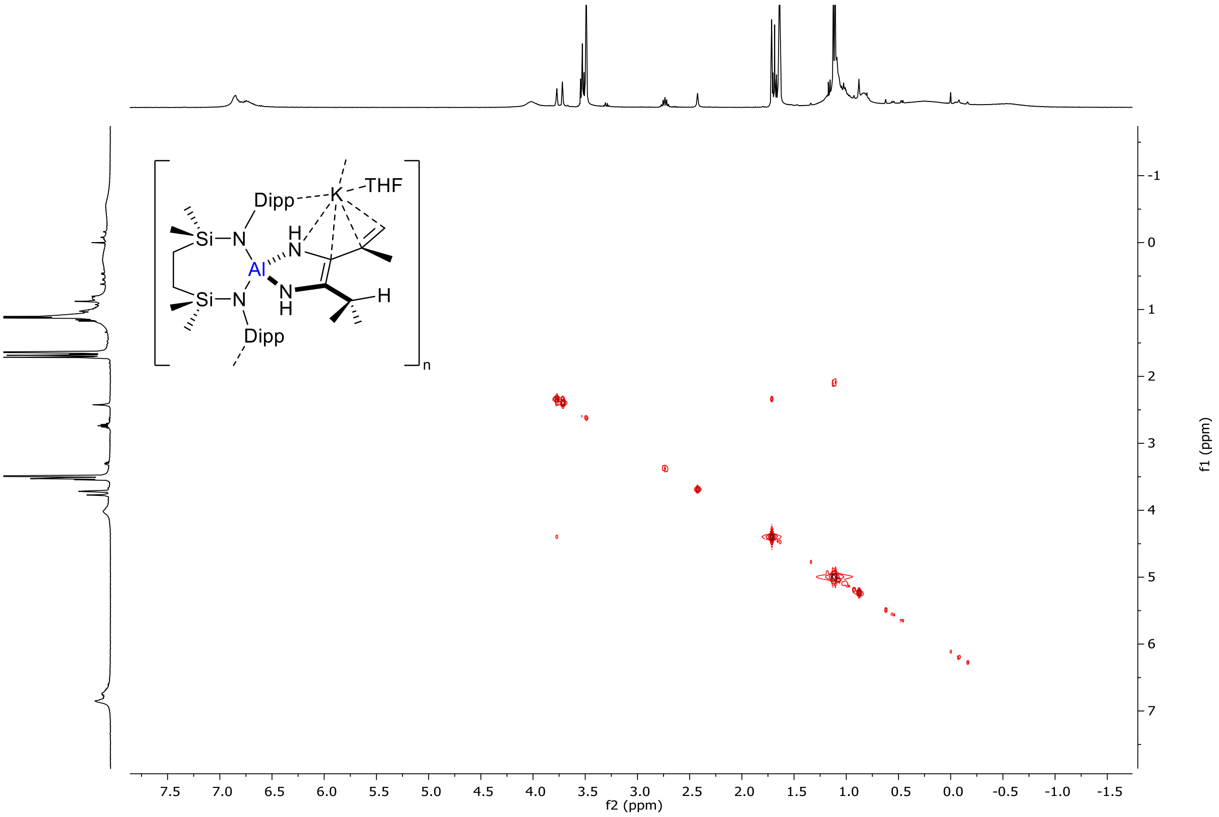

X-ray crystallography

Data for all structures were obtained using an Agilent SuperNova instrument and a Cu-Kα source. All experiments were conducted at 150 K, solved using SHELXT[1] and refined using SHELXL[2] via the Olex2[3] interface. Refinements were largely uneventful. Where disorder prevailed, appropriate distance and ADP restraints were included, in these regions, to assist convergence. Only additional, noteworthy, points follow.

In addition to one molecule of the K/Al complex, the asymmetric unit in the structure of **2** was seen to play host to half of a hexane moiety (proximate to a crystallographic inversion centre which serves to generate the remainder). Disorder was modelled for the THF ligand containing O1 (60:40), the THF based on O2 (50:50) and atoms C15-16 (83:17).

The gross structure of **3** is dominated by 1-D polymers that propagate along the *b* axis. The hydrogen atoms attached to C13 were located and refined subject to being at a distance of 0.98 Å from the parent atom.

The asymmetric unit in the structure of **4** is a monomer. This motif is the basis for 1-D polymers dominating the gross structure. C40 and C41 were treated for a 50:50 disorder. The hydrogen atoms attached to the nitrogens (as well as the those attached to C37 and to C14) were located and refined at a distance of 0.96 Å from the relevant parent atoms.

Crystallographic data for all compounds have been deposited with the Cambridge Crystallographic Data Centre as supplementary publications CCDC 2364568-2364571 for **1** - **4**, respectively. Copies of these data can be obtained free of charge on application to CCDC, 12 Union Road, Cambridge CB2 1EZ, UK [fax(+44) 1223 336033, e-mail: deposit@ccdc.cam.ac.uk.

Table S1: Data collection parameter and refinement details for **1**, **2**, **3** and **4**.

Manuscript Identifier	**1**	**2**	**3**	**4**
Empirical formula	$C_{98}H_{134}Al_2K_2N_8Si_4$	$C_{57}H_{87}AlKN_4O_2Si_2$	$C_{35}H_{59}AlKN_3Si_2$	$C_{42}H_{72}AlKN_4OSi_2$
Formula weight	1668.64	982.56	644.11	771.29
Crystal system	monoclinic	triclinic	orthorhombic	monoclinic
Space group	$P2_1/c$	*P*-1	*Pbca*	$P2_1/c$
a/ Å	12.3727(2)	13.0244(2)	12.2879(1)	10.4318(1)
b/ Å	43.9961(5)	14.0474(3)	18.2457(2)	21.6718(1)
c/ Å	18.1544(2)	19.0196(3)	33.9082(4)	20.7534(1)
α/ °	90	98.921(2)	90	90
β/ °	104.923(1)	104.173(2)	90	103.608(1)
γ/ °	90	115.962(2)	90	90
U/ $Å^3$	9549.1(2)	2892.9(1)	7602.26(14)	4560.13(6)
Z	4	2	8	4
ρ_{calc}/ g cm^{-3}	1.161	1.128	1.126	1.123
μ/ mm^{-1}	1.902	0.190	2.238	1.963
F(000)	3592.0	1066.0	2800.0	1680.0
Crystal size/ mm^3	0.108 × 0.064 × 0.049	0.259 × 0.211 × 0.156	0.237 × 0.101 × 0.048	0.273 × 0.227 × 0.133
2θ range for data collection/°	7.662 to 146.136	3.382 to 52.288	8.888 to 146.13	8.16 to 146.536
Index ranges	$-15 \leq h \leq 15$, $-54 \leq k \leq 50$, $-22 \leq l \leq 19$	$-16 \leq h \leq 15$, $-17 \leq k \leq 17$, $-18 \leq l \leq 23$	$-11 \leq h \leq 15$, $-22 \leq k \leq 22$, $-41 \leq l \leq 41$	$-12 \leq h \leq 12$, $-26 \leq k \leq 26$, $-25 \leq l \leq 23$
Reflections collected	93702	40308	99245	64757
Independent reflections, R_{int}	19006, 0.0606	11522, 0.0280	7584, 0.0702	9125, 0.0335
Data/restraints/parameters	19006/0/1055	11522/345/727	7584/3/405	9125/74/520
Goodness–of–fit on F^2	1.023	1.017	0.948	1.028
Final *R*1, *wR*2 [I>=2σ(I)]	0.0456, 0.1053	0.0439, 0.1163	0.0358, 0.0929	0.0358, 0.1001
Final *R*1, *wR*2 [all data]	0.0630, 0.1139	0.0477, 0.1198	0.0441, 0.0987	0.0386, 0.1027
Largest diff. peak/hole/ e $Å^{-3}$	0.34/–0.24	0.66/–0.50	0.35/–0.22	0.31/–0.33

References

1. Dolomanov, O. V.; Bourhis, L. J.; Gildea, R. J.; Howard, J. A. K.; Puschmann, H., *J. Appl. Cryst.* **2009**, *42*, 339-341.
2. Sheldrick, G. M., *Acta Cryst*. **2015**, *A71*, 3-8.
3. Sheldrick, G. M., *Acta Cryst*. **2015**, *C71*, 3-8.
